# Enhancing dose conformity in head and neck intensity‐modulated proton therapy using a novel dynamic multi‐leaf collimator strategy

**DOI:** 10.1002/mp.70355

**Published:** 2026-02-19

**Authors:** Yushi Wakisaka, Yuki Tominaga, Yuya Miyasaka, Robabeh Rahimi, Keith M Furutani, Mayu Nakata, Takeo Iwai, Teiji Nishio

**Affiliations:** ^1^ Department of Radiotherapy Medical Co. Hakuhokai, Osaka Proton Therapy Clinic Osaka Japan; ^2^ Medical Physics Laboratory Division of Health Science Graduate School of Medicine The University of Osaka Osaka Japan; ^3^ Department of Heavy Particle Medical Science Yamagata University Graduate School of Medical Science Yamagata Japan; ^4^ Department of Radiation Oncology University of Maryland School of Medicine Baltimore Maryland USA; ^5^ Division of Medical Physics Department of Radiation Oncology Mayo Clinic Florida Jacksonville Florida USA; ^6^ Department of Carbon Ion Radiotherapy Graduate School of Medicine The University of Osaka Osaka Japan

**Keywords:** dynamic collimation, multi leaf collimator, proton therapy

## Abstract

**Background:**

Proton pencil beam scanning (PBS) enables highly conformal dose distributions; however, its lateral dose fall‐off (penumbra) can be compromised by the use of range shifters (RSs) and increased air gaps. In PBS for head and neck regions, where critical organs at risk (OARs) are frequently adjacent to the target, penumbra degradation may lead to increased OAR doses or suboptimal target coverage. The integration of a dynamic multi‐leaf collimator (dMLC), which adjusts leaf positions at each energy layer, has been shown to improve dose conformity in single‐field uniform dose (SFUD) delivery. In parallel, intensity‐modulated proton therapy (IMPT) offers enhanced dose shaping over SFUD by modulating beam intensity across multiple fields and does not require a single beam to encompass the entire target volume, providing greater flexibility in utilizing dMLC capabilities.

**Purpose:**

This study integrates dMLC into IMPT for head and neck cancer and proposes a novel leaf positioning strategy. We evaluate the dosimetric impact of this approach and assess its potential clinical benefits in terms of target coverage and OAR sparing.

**Methods:**

Treatment plans were retrospectively created for five patients with head and neck cancer. For each patient, IMPT plans with three beam angles were generated using three techniques: (1) uncollimated PBS, (2) dMLC_cover_, in which the MLC encloses the target cross‐section at each energy layer, and (3) dMLC_block_, in which the MLC actively blocks OARs and their distal regions. Dose‐volume histogram (DVH) metrics for the clinical target volume (CTV) and OARs were evaluated, including a total of 21 perturbed scenarios that combined ± 2 mm setup uncertainties (7 scenarios) and ± 3.5% range uncertainties (3 scenarios). The accuracy of dose calculations was validated by comparing calculated and measured lateral dose distributions at the isocenter plane in water using two‐dimensional gamma analysis with a 2%/2 mm criterion.

**Results:**

The integration of dMLC with IMPT significantly reduced the dose to surrounding OARs while maintaining comparable target coverage and robustness relative to uncollimated PBS. Notably, dMLC_block_ demonstrated an enhanced dose‐sparing effect than dMLC_cover_, particularly for OARs surrounded by the target. While maintaining comparable CTV D98% across three techniques, dMLC_cover_ achieved the greatest reduction in the mean doses to the eyeballs and optic nerves, as well as in the D2% to the optic chiasm, brain, and brainstem in most cases. The gamma passing rate between calculated and measured doses for dMLC_block_ exceeded 95% for all beams, confirming the accuracy of dose calculations involving complex leaf positions.

**Conclusions:**

The combination of IMPT and dMLC provides notable dosimetric advantages, supporting its potential for clinical applications. Further validation across a broader range of cases is necessary to comprehensively assess its efficacy and safety, particularly with respect to leaf positioning accuracy and potential variations in biological effectiveness.

## 1 INTRODUCTION

Head and neck cancer accounts for approximately 5% of newly diagnosed cancer cases annually.^1^ Standard treatment typically involves a multidisciplinary approach incorporating surgery, radiation therapy, and chemotherapy.^2^ While conventional X‐ray radiotherapy has historically been the predominant modality^3^ proton therapy (PT), with its superior dose conformity and normal tissue sparing, has gained increasing interest.^4^, ^5^

The head and neck region presents considerable anatomical complexity, with numerous organs‐at‐risk (OARs) in close proximity to the target, often compromising optimal dose delivery. Insufficient target coverage due to these constraints may contribute to local recurrences.^6^ Therefore, enhancing proton dose distribution to improve both tumor coverage and OAR sparing is a priority in head and neck cancer treatment.

Traditionally, passive scattering has been the primary technique in PT, relying on collimators and boluses to shape the beam laterally and distally.^7^ More recently, pencil beam scanning (PBS) has been widely adopted due to its capability for highly conformal dose delivery and elimination of physical apertures.^8^ However, PBS exhibits less favorable lateral dose fall‐off, penumbra, compared to passive scattering, especially at shallow depths.^9^ The penumbra degradation is further exacerbated by the use of range shifters (RSs) or increasing air gaps.^10^

The MELTHEA V PT system (Hitachi Ltd., Tokyo, Japan) incorporates a multipurpose nozzle capable of switching between passive scattering and PBS modes, and supports PBS delivery with a multi‐leaf collimator (MLC).^11^ Prior studies have demonstrated using an MLC with PBS can sharpen the lateral penumbra.^12^ Further improvements have been demonstrated using a dynamic MLC (dMLC), which adjusts the leaf configuration layer‐by‐layer; however, such studies have so far focused exclusively on single‐field uniform dose (SFUD) techniques.^13^

As dynamic collimation techniques in scanning PT, the Dynamic Collimation System, which shapes the target for each energy layer using two movable blades^14^, ^15^, ^16^ and the Dynamic Aperture integrated into the Mevion S250i Hyperscan system^17^, ^18^, ^19^ have been developed, both of which have been shown to improve penumbra and reduce normal tissue dose. However, these reports have also been limited to SFUD.

Intensity‐modulated proton therapy (IMPT), which delivers modulated beam intensities from multiple directions to achieve a homogeneous cumulative dose distribution, has demonstrated superior dose conformity compared to SFUD.^20^ Unlike SFUD, IMPT does not require a single beam to cover the entire target volume, providing greater flexibility for integrating dynamic collimation. To our knowledge, however, the application of dMLC in IMPT has not yet been explored.

In this study, we integrate dMLC into IMPT planning for head and neck cancer and propose a novel leaf positioning strategy. We evaluate the resulting dosimetric improvements and assess potential clinical benefits with respect to target coverage and OAR sparing, supported by experimental validation of dose calculation accuracy.

## 2 METHODS AND MATERIALS

### 2.1 Machine specifications

The MELTHEA V system comprises a synchrotron‐based accelerator, a rotating gantry, and a multipurpose nozzle. The nozzle supports both passive scattering and PBS modes and allows for the insertion of an upstream MLC and a downstream RS. The nozzle position is adjustable within a range of 250–560 mm from the isocenter.^12^

The accelerator delivers 92 beam energies ranging from 70.7 to 235.0 MeV, corresponding to a water‐equivalent length (WEL) of 40–340 mm. For shallow targets (depths less than 40 mm WEL), an RS with 60–66 mm WEL thickness is required. Without an RS, the air spot size (*σ*) at the isocenter plane is 3.2 mm at maximum energy and 9.9 mm at minimum energy. The use of an RS further increases the air spot size depending on the nozzle‐to‐isocenter distance.

The MLC consists of 54 opposing leaf pairs made of iron and incorporates a tongue‐and‐groove design to minimize interleaf leakage. Each leaf has a width of 3.75 mm and a thickness of 140 mm. The maximum opening per pair is 75 mm, yielding a rectangular maximum field size of 150 mm (parallel to leaf motion) by 200 mm (perpendicular to leaf motion). Note that all dimensions described here are physical dimensions.

### 2.2 Patient selection and dataset

Five patients with head and neck cancer who underwent radiotherapy at the East Japan Heavy Ion Center between May 2022 and February 2024 were retrospectively analyzed in this study. Patient characteristics are summarized in Table 1. Ethical approval was obtained from the institutional review board (ethics committees) of Yamagata University Hospital (No. 2024–130) and Medical Corporation, Hakuhokai, Ako Central Hospital (No. 20241207).

**TABLE 1:** Patient characteristics and beam information.

#Patient	Diagnosis	Location	TNM	CTV volume [cc]	#Beam	Gantry angle [degree]	Collimator angle [degree]
Patient 1	Adeno‐carcinoma	Left maxillary sinus	T3N0M0	157	Beam 1	0	0
					Beam 2	90	90
					Beam 3	330	90
Patient 2	Adenoid cystic carcinoma	Right nasal cavity	T2N0M0	188	Beam 1	10	90
					Beam 2	60	90
					Beam 3	300	0
Patient 3	Malignant melanoma	Right nasal cavity	T4N0M0	104	Beam 1	0	90
					Beam 2	25	90
					Beam 3	330	0
Patient 4	Sarcoma	Left nasal cavity	T2N0M0	104	Beam 1	0	0
					Beam 2	25	90
					Beam 3	335	90
Patient 5	Malignant melanoma	Right nasal cavity	T4N0M0	130	Beam 1	0	90
					Beam 2	20	0
					Beam 3	340	0

For each patient, fused computed tomography (CT) and magnetic resonance imaging (MRI) datasets were used to delineate the gross tumor volume (GTV) and clinical target volume (CTV), as determined by radiation oncologists. The CT and MRI images were acquired with a 2‐mm slice thickness, and the MRI protocol included T1‐weighted and T2‐weighted sequences. The CTV volumes for each patient are listed in Table 1. OARs, including the brain, brainstem, optic chiasm, optic nerves, and eyes, were delineated according to the guideline^21^. PT treatment plans were generated retrospectively based on the CT images and associated structure sets for evaluation purposes.

### 2.3 Leaf positioning for dynamic MLC

**FIGURE 1:**
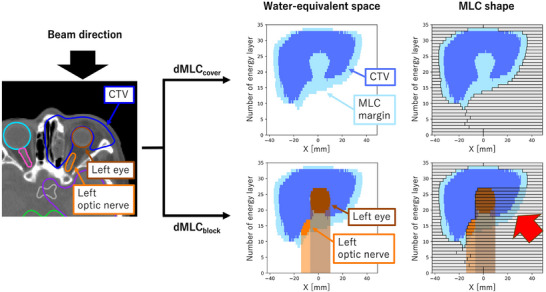
Procedure for calculating leaf positions for dMLC_cover_ and dMLC_block_ in Beam 1 of Patient 1. The blue, light blue, brown, and orange‐filled areas in the 2D plot on the right represent the cross‐sections of the CTV, MLC margin, left eye, and left optic nerve at each energy layer, respectively. Note that the vertical axis represents the direction of the energy layers. The target regions indicated by the red arrows are blocked by the beam from this direction but are compensated by beams from other directions.

dMLC requires adjusting the leaf positions for each energy layer. Two dMLC configurations were implemented and evaluated using the scripting interface of the RayStation 10A treatment planning system (TPS; RaySearch Laboratories, Stockholm, Sweden). Figure 1 illustrates the procedure for calculating leaf positions for beam 1 of Patient 1.

In the first configuration of dMLC, termed dMLC_cover_, the MLC leaves are positioned to enclose the cross‐sectional projection of the target volume at each energy layer. This is accomplished by converting the target structure into water‐equivalent space and adding an MLC margin to its cross‐section at the Bragg peak depth. The approach follows a previous study^13^ with the MLC margin defined as 0.5*σ* (where *σ* represents the in‐medium spot size at the corresponding energy layer).

The second configuration of dMLC, termed dMLC_block_, includes an additional OAR‐blocking algorithm. In this method, both the target and OARs are converted into water‐equivalent space, and MLC leaves are positioned to block the OAR and its distal region from the beam's eye‐view. Although this method leads to intentional underirradiation from certain angles, as illustrated by the red arrows in Figure 1, these regions are compensated by beams from alternative angles.

Water‐equivalent path lengths for each structure were computed via ray tracing from the scanning nozzle to the relevant CT voxels, using the CT number‐to‐stopping power conversion table embedded in the TPS.

### 2.4 Treatment planning

Multi‐field optimized IMPT plans were generated for each patient using the TPS with three beam angles. The selected gantry angles and collimator rotation angles are listed in Table 1 and were chosen to ensure that no portion of the target was blocked by the dMLC_block_ configuration in any beam. This was achieved by using the scripting function of the TPS to delineate the blocked regions as beam‐specific contours, followed by iterative trial‐and‐error adjustments to ensure that these regions did not overlap within the target volume. For all plans, the RS thickness was fixed at 60 mm, and the air gap, defined as the minimum distance between the RS and the patient surface, was set to 30 mm.

Three treatment techniques were evaluated: (1) uncollimated PBS, (2) dMLC_cover_, and (3) dMLC_block_. Beam spots were arranged on a hexagonal grid with a spacing of 0.75*σ*, and a lateral margin of 1.0*σ* was applied (i.e., spots were placed 1.0*σ* beyond the lateral boundary of the target). In the dMLC_block_ plans, spots that overlapped the MLC leaf projection by more than 0.5*σ* were excluded. Detailed MLC shapes and spot placements are shown in Supplementary Materials Figure 1. Since the pencil beam algorithm does not accurately model MLC‐modulated beams, dose calculations were performed using the simplified Monte Carlo algorithm implemented in the TPS.^12^ A sampling history of 10 000 ions per spot was used during the optimization process, and the statistical uncertainty for the final Monte Carlo dose calculation was set to 0.5%.^22^ The dose calculation grid size was uniformly set to 2 mm.

The prescription dose was 74 GyRBE in 37 fractions,^23^ prescribed to the median dose of the CTV, with planning goals of D98% of the CTV received at least 95% of the prescription dose and D2% remained below 107%.^24^ Dose constraints for OARs were applied using equivalent uniform dose (EUD)‐based criteria^25^

Given the sensitivity of IMPT to range and setup uncertainties, worst‐case robust optimization was performed.^26^ Robustness settings included a positional uncertainty of ± 2.0 mm and a range uncertainty of ± 3.5%.^27^, ^28^

### 2.5 Dose evaluation metrics

For the three treatment plans (i.e., PBS, dMLC_cover_, and dMLC_block_), dose‐volume histogram (DVH) indices were evaluated, including CTV D98% and D2%; mean dose (Dmean) to the eyes and optic nerves; and D2% to the optic chiasm, brain, and brainstem.

To assess the setup and range uncertainties, perturbed dose distributions were calculated for 21 scenarios, comprising seven positional shifts (0 mm and ± 2 mm shifts in cranialcaudal, anteriorposterior, and left‐right directions) and three range uncertainty settings (0% and ± 3.5%). DVH indices under each scenario were analyzed for all three planning techniques.

Statistical comparisons of DVH indices across the three techniques (i.e., PBS, dMLC_cover_, and dMLC_block_) were performed using the two‐sided Wilcoxon signed‐rank test, with multiple comparisons adjusted using the Holm correction. A *p*‐value of < 0.05 was considered statistically significant.

### 2.6 Dose verification

Dose calculation accuracy was evaluated by comparing calculated and measured lateral dose distributions for 18 beams in total—three beams each from the uncollimated PBS and dMLCblock treatment plans for all five patients.

First, dose distributions for each beam in a water medium were recalculated using the TPS, and corresponding 2D dose profiles at the isocenter plane were extracted. Measurements of 2D dose distribution at the same plane were then performed using a cross‐calibrated OCTAVIUS Detector 729 (2D‐array, PTW, Freiburg, Germany) in combination with a solid water phantom (Tough Water; Kyoto Kagaku, Kyoto, Japan). The OCTAVIUS system consists of 729 ionization chambers with a sensitive volume of 0.075 cc (5 × 5 × 3 mm^3^) arranged in a 2D array with 10 mm spacing^12^ Additionally, for all beams of Patient 1, supplementary measurements were performed using radiochromic film that had been pre‐calibrated using a dose–response curve.

Because the PT system used in this study does not support dMLC functionality, that is leaf shape modulation across energy layers, each energy‐split beam was measured individually, and the corresponding dose distributions were subsequently summed to obtain the total dose.

Finally, 2D gamma analysis was conducted to assess agreement between calculated and measured dose distributions using in‐house software based on the Python pymedphys library. A 10% dose threshold was applied, using gamma criteria of a 2% dose difference and a 2 mm distance‐to‐agreement.^29^ A gamma passing rate ≥95% was considered acceptable.^30^

## 3 RESULTS

### 3.1 Dose distribution and DVH analysis

**FIGURE 2:**
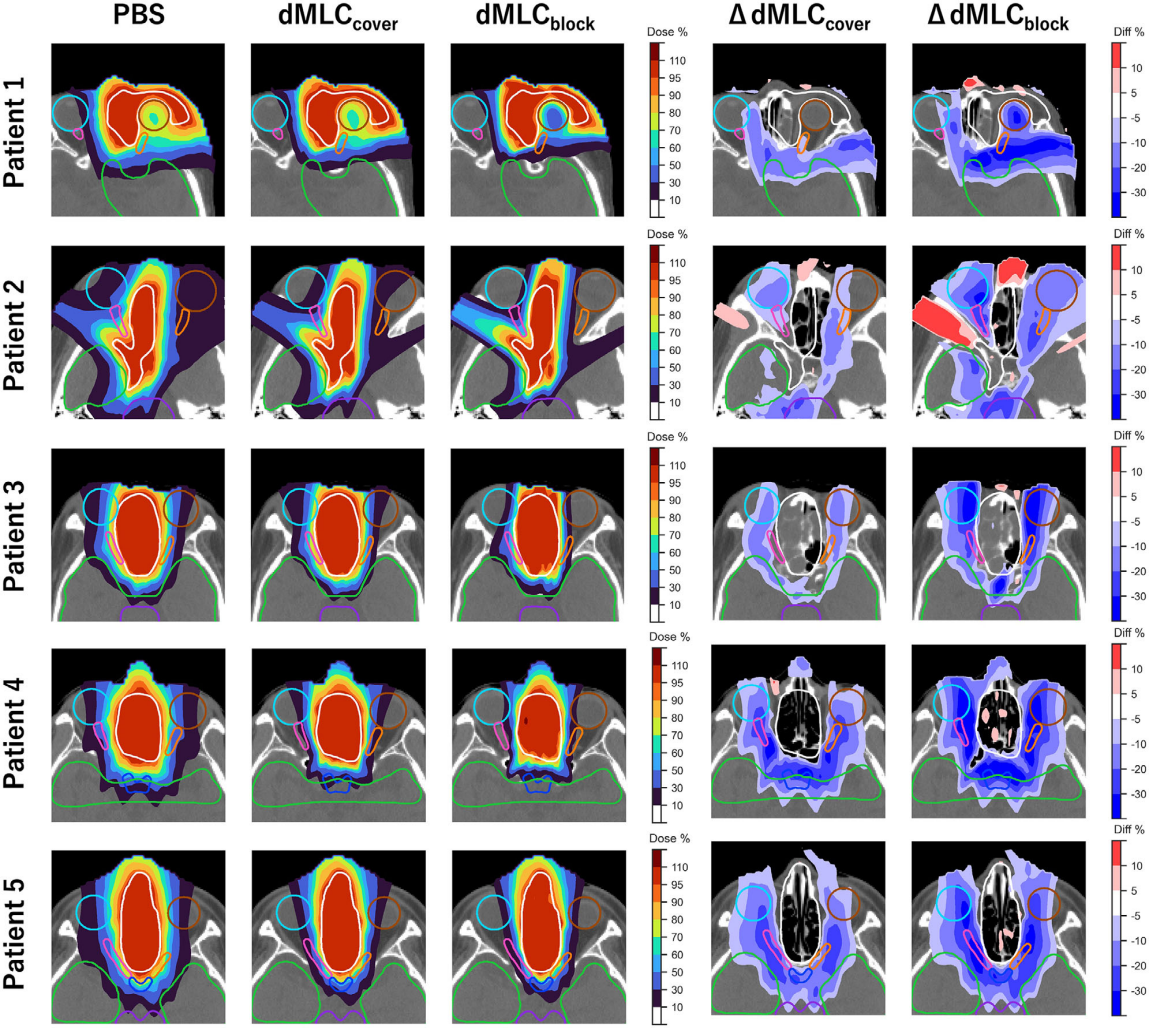
Dose distributions for the three treatment plans of uncollimated PBS, dMLC_cover_, and dMLC_block_ (left three columns), and the dose differences compared to uncollimated PBS (right two columns). The white, brown, cyan, orange, pink, green, and purple contours represent the CTV, left eye, right eye, left optic nerve, right optic nerve, brain, and brainstem, respectively.

Figure 2 shows the dose distributions for the three treatment plans—uncollimated PBS, dMLC_cover_ and dMLC_block_—as well as dose differences relative to uncollimated PBS. For adjacent OARs that were not surrounded by the target (e.g., the brain and brainstem), MLC_cover_ reduced the dose compared to uncollimated PBS. However, for OARs that were surrounded by the target (e.g., the left eye in Patient 1 and the right optic nerve in Patient 2), the dose reduction with MLC_cover_ was limited, while MLC_block_ achieved a more substantial reduction.

**FIGURE 3:**
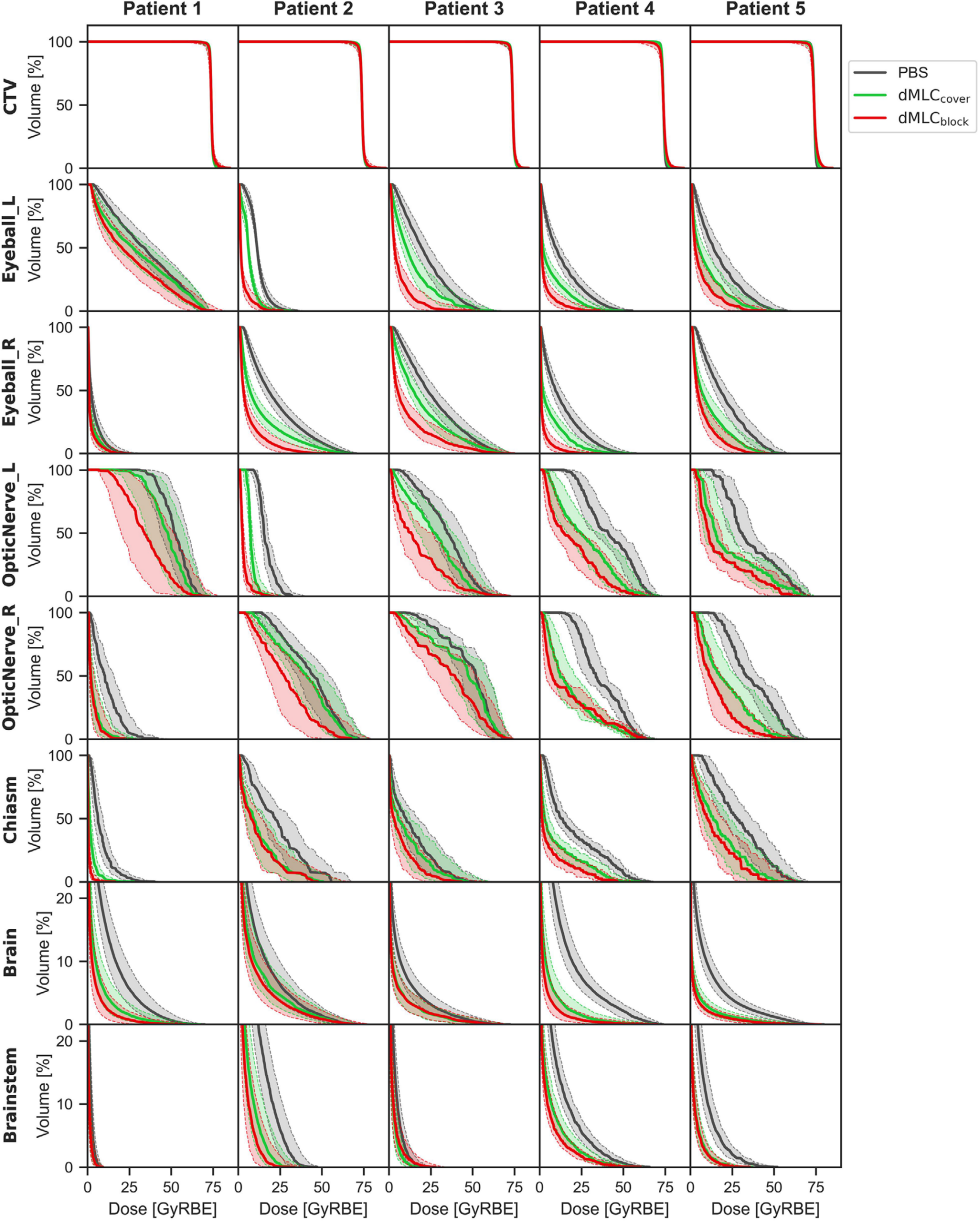
DVH for the three treatment plans of uncollimated PBS (gray), dMLC_cover_ (green), and dMLC_block_ (red). The bold line and the shaded area represent the nominal dose and the error scenario doses, respectively.

**FIGURE 4:**
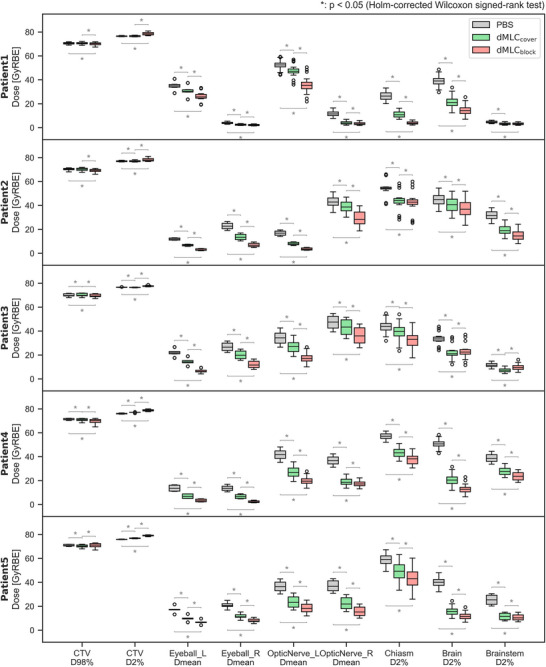
Dose indices for the three treatment plans of uncollimated PBS (gray), dMLC_cover_ (green), and dMLC_block_ (red) for all evaluated error scenarios. The asterisks represent the significant differences (*p* < 0.05) between treatment plans.

Figures 3 and 4 present the DVH curves and dose indices for all evaluated uncertainty scenarios. CTV coverage remained robust across all plans, with comparable D98% and D2% values. Doses to most OARs were significantly lower with MLC_cover_ than with uncollimated PBS, and were further reduced with MLC_block_ compared to MLC_cover_. The only exception was in Patient 3, where the D2% to the brain and brainstem was slightly higher with MLC_block_ than with MLC_cover_, although it remained within clinically acceptable limits.

### 3.2 Dose verification

**FIGURE 5:**
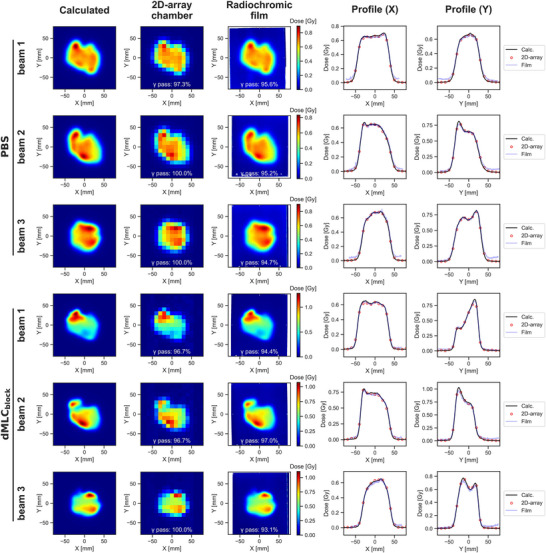
The calculated and measured dose distributions at the isocenter plane for all beams of Patient 1. The upper and lower three rows show the results for uncollimated PBS and dMLC_block_, respectively. The left three columns display 2D dose distributions with gamma passing rates (2%/2 mm, 10% threshold), while the right two columns show line dose profiles at *Y* = 0 and *X* = 0.

Figure 5 displays the calculated and measured lateral dose distribution at the isocenter plane for Beam 1 of Patient 1. The measured doses closely reproduced the calculated distributions for both uncollimated PBS, characterized by strong intensity modulation, and dMLC_block_, which featured a steeper lateral penumbra.

Table 2 summarizes the gamma passing rates for all verified beams, evaluated using the 2%/2 mm criterion. The mean gamma passing rates were 99.5% for uncollimated PBS and 98.9% for dMLC_block_. Although dMLC_block_ showed slightly lower gamma passing rates than uncollimated PBS across all beams, all values exceeded the 95% threshold, confirming the accuracy of the dose calculations even with complex leaf configurations.

**TABLE 2:** Gamma passing rates for uncollimated PBS and dMLC_block_ using the 2%/2 mm criterion with a 10% cutoff threshold. Values in each cell represent the gamma passing rates for beam 1, beam 2, and beam 3, respectively.

	Patient 1	Patient 2	Patient 3	Patient 4	Patient 5
PBS	97.3, 100, 100%	100, 100, 100%	97.9, 100, 100%	100, 100, 100%	100, 100, 100%
dMLC_block_	96.7, 96.7, 100%	100, 100, 100%	97.9, 100, 100%	100, 97.5, 100%	100, 100, 100%

## 4 DISCUSSION

This study evaluated improvement in dose distribution achieved by integrating dMLC into IMPT for head and neck cancer. In all five cases analyzed, dMLC maintained target coverage compared to uncollimated PBS while substantially reducing the dose to surrounding OARs.

Among the two dMLC configurations, dMLC_block_, which actively blocked OARs using leaf positioning, demonstrated superior dose‐sparing effects compared to dMLC_cover_, which passively enclosed the target cross‐section at each energy layer. The advantage of dMLC_block_ was particularly evident for OARs embedded within or adjacent to the target, whereas dMLC_cover_ provided limited benefit for such cases. This level of sparing was not achieved with dMLC applied to SFUD plans.^13^, ^31^ The only exception was a slight increase in D2% to the brain and brainstem in Patient 3 when using dMLC_block_ relative to dMLC_cover_. This is likely attributable to the fact that, in this case, the beams contributing to the dose in the brain, particularly near the brainstem, were largely limited to those delivered at gantry angles close to 0°, resulting in a slight increase in the dose to the distal region encompassing the brain and brainstem. Nevertheless, the dose remained within clinically acceptable limits.

We evaluated OAR doses from the perspective of clinically relevant adverse events. For example, a Dmean ≥ 35 Gy to the eyeball has been associated with an increased risk of severe late toxicities, including corneal ulcer, xerophthalmia, glaucoma, and retinopathy.^32^ In Patient 1, whose left eyeball was located extremely close to the CTV, only the dMLC_block_ plan maintained the dose below this threshold across all perturbation scenarios. Additionally, a Dmean ≥ 12 Gy has been reported to correspond to a > 25% incidence of ocular pain.^33^ Among 10 eyeballs in the 5 patients, this threshold was exceeded in 9 eyes for uncollimated PBS, in 5 eyes with dMLC_cover_, and only 1 eye with dMLC_block_.

For the optic nerves, previous studies have reported that a Dmean > 50 Gy leads to significant deterioration of visual functions, including visual acuity, visual field, contrast sensitivity, and visually evoked potential latency.^34^, ^35^ Across all perturbation scenarios, the number of optic nerves (out of 10 in 5 patients) exceeding this threshold was three with uncollimated PBS, two with dMLC_cover_, and none with dMLC_block_. Regarding the optic chiasm, maintaining the dose at ≤ 55 Gy has been suggested to limit the risk of optic neuropathy or chiasmal injury to 3%.^36^ In Patients 4 and 5, where the chiasm was located near the target, threshold exceedances were observed only with uncollimated PBS; both dMLC_cover_ and dMLC_block_ maintained doses below the recommended limit.

For doses to the brain and brainstem, QUANTEC recommends limits of ≤ 60 Gy and ≤ 54 Gy, respectively.^36^ All three irradiation approaches evaluated in this study met these dose constraints.

The CTV D98% varied slightly among the three techniques —uncollimated PBS, dMLC_cover_, and dMLC_block_— depending on the patient; however, the differences were minor, and all plans met the planning objective of delivering at least 95% of the prescribed dose. In contrast, although the CTV D2% met the upper dose constraint of ≤107% for all patients, the dMLC_block_ technique consistently achieved the highest values among the three approaches. These results indicate that while dMLC_block_ offers improved OAR sparing, it is associated with a modest reduction in dose homogeneity within the CTV, though the overall impact remained clinically acceptable.

While the use of dMLC improves dose conformity, it raises concerns regarding robustness. However, the application of worst‐case robust optimization in this study successfully preserved target dose robustness across all scenarios. A uniform MLC margin of 0.5*σ* was applied, consistent with previous study^13^ that did not incorporate robustness considerations. Given that optimal MLC margins may vary under robust optimization, further investigation is warranted to determine whether smaller or adaptive margins could improve plan quality without compromising robustness. Nonetheless, our findings demonstrate that a 0.5*σ* margin is sufficient to maintain robustness comparable to that of uncollimated PBS plan.

The gantry and collimator rotation angles were selected to ensure that no portions of the target were blocked by the dMLC_block_ configuration in any beam. Those same angles were used for the uncollimated PBS and dMLC_cover_ plans to ensure consistency. Although optimal beam angles can vary between patients and planning techniques, this study did not explore angle optimization because the TPS does not provide a systematic method for optimizing these parameters. As appropriate gantry and collimator selections currently rely on a trial‐and‐error process, future work aimed at developing or integrating angle optimization strategies is warranted. In the five cases analyzed, full target coverage was achieved using three beams; however, more complex cases may require additional beams.

Although Patient 1 exhibited a target geometry that differed from the other cases, similar dosimetric trends—namely, maintained CTV coverage and improved OAR sparing with dMLCblock—were consistently observed across the remaining patients. The observed benefits were therefore not driven by a single case but reflected a common tendency within the evaluated cohort. Further studies with a larger and more diverse patient population are required to confirm the generalizability of these findings.

As this study was an insilico study, the effectiveness of dMLC was evaluated only theoretically. The PT system used does not currently support dMLC‐based irradiation, and therefore, further investigations following hardware implementation are necessary to assess actual dynamics and potential treatment time implications.

For dose verification, energy‐layer‐split beams were measured individually and then summed to approximate the total dose, a process that, while time‐consuming, provided verification equivalent to what would be expected with a dMLC‐equipped system. To assess whether the OCTAVIUS Detector 729 —whose detector elements have a relatively large active volume— is suitable for evaluating steep penumbra, supplementary radiochromic film measurements were performed for all beams of one representative patient. As shown in Figure 5, the film measurements reproduced the planned dose in most regions, including the penumbra, and demonstrated good agreement with the OCTAVIUS 729 measurements, indicating that verification using the OCTAVIUS 729 is sufficiently reliable. Although radiochromic film measurements were performed only for Patient 1 as a representative case, similar agreement between calculated and measured dose distributions was consistently observed for all other patients based on 2D array measurements, with gamma passing rates exceeding 95% for all beams. As summarized in Table 2, dMLC_block_ exhibited gamma passing rates that were slightly lower or comparable to those of uncollimated PBS across all beams, which is considered to be partly attributable to the reduced number of evaluation points resulting from the smaller irradiation fields.

Nevertheless, despite the complexity of leaf modulation in dMLC_block_, all gamma passing rates exceeded 95% using the 2%/2 mm criterion, confirming the accuracy of the dose calculation. The high agreement between calculated and measured doses may, in part, be due to the use of a simplified Monte Carlo algorithm in the TPS^12^

The dose calculation results for IMPT with dMLC demonstrated significantly improved dose conformity, which was corroborated by experimental verification, supporting its feasibility for clinical implementation. To enable clinical adoption, it will be necessary to develop a real‐time leaf position monitoring system and to assess the impact of potential leaf positioning inaccuracies on delivered dose.

Furthermore, prior studies have indicated that proton beam collimation can modify the dose‐averaged linear energy transfer (LET), which may influence biological effectiveness.^37^ As such, integrating biological dose evaluation into treatment planning may be required to fully establish the clinical value of dMLC‐based IMPT. Ultimately, validation across a broader patient cohort and a range of anatomical scenarios will be essential to comprehensively assess the dosimetric, biological, and operational implications of this technique, laying the groundwork for dMLC as a next‐generation clinical irradiation strategy in PT.

## 5 CONCLUSION

This study demonstrated that integrating a dynamic MLC, dMLC, with IMPT can reduce the dose to OARs adjacent to target, while maintaining target coverage. Notably, the dMLC_block_ strategy which actively blocks OARs through dynamic leaf positioning, achieved greater dose sparing than dMLC_cover_, particularly for OARs that are surrounded by the target volume.

Dose verification using gamma analysis with a 2%/2 mm criterion confirmed the accuracy of the calculated dose distributions, with all gamma passing rates exceeding 95%. These results support the feasibility of dMLC‐based IMPT for clinical implementation. However, further evaluation of leaf positioning accuracy, delivery dynamics, and potential biological effects will be essential to fully establish the efficacy and safety of this technique in routine clinical applications.

## CONFLICT OF INTEREST STATEMENT

The authors declare no conflicts of interest.
